# Development of a framework for the co-production and prototyping of public health interventions

**DOI:** 10.1186/s12889-017-4695-8

**Published:** 2017-09-04

**Authors:** Jemma Hawkins, Kim Madden, Adam Fletcher, Luke Midgley, Aimee Grant, Gemma Cox, Laurence Moore, Rona Campbell, Simon Murphy, Chris Bonell, James White

**Affiliations:** 1 Centre for the Development and Evaluation of Complex Interventions for Public Health Improvement (DECIPHer), Cardiff University, Cardiff, CF10 3BD UK; 2 Centre for Trials Research, Cardiff University, Cardiff, CF14 4YS UK; 3 Y Lab, Cardiff University, Cardiff, CF10 3AT UK; 4 Public Health Wales, Cardiff, CF10 4BZ UK; 5 Medical Research Council /Chief Scientist Office Social and Public Health Sciences Unit, University of Glasgow, Glasgow, G2 3QB UK; 6 Centre for the Development and Evaluation of Complex Interventions for Public Health Improvement (DECIPHer), University of Bristol, Bristol, BS8 2PS UK; 7 London School of Hygiene and Tropical Medicine, London, WC1H 9SH UK

**Keywords:** Intervention development, Public health, Co-production, Prototyping, Transdisciplinary action research, Drug prevention, Adolescence

## Abstract

**Background:**

Existing guidance for developing public health interventions does not provide information for researchers about how to work with intervention providers to co-produce and prototype the content and delivery of new interventions prior to evaluation. The ASSIST + Frank study aimed to adapt an existing effective peer-led smoking prevention intervention (ASSIST), integrating new content from the UK drug education resource Talk to Frank (www.talktofrank.com) to co-produce two new school-based peer-led drug prevention interventions. A three-stage framework was tested to adapt and develop intervention content and delivery methods in collaboration with key stakeholders to facilitate implementation.

**Methods:**

The three stages of the framework were: 1) Evidence review and stakeholder consultation; 2) Co-production; 3) Prototyping. During stage 1, six focus groups, 12 consultations, five interviews, and nine observations of intervention delivery were conducted with key stakeholders (e.g. Public Health Wales [PHW] ASSIST delivery team, teachers, school students, health professionals). During stage 2, an intervention development group consisting of members of the research team and the PHW ASSIST delivery team was established to adapt existing, and co-produce new, intervention activities. In stage 3, intervention training and content were iteratively prototyped using process data on fidelity and acceptability to key stakeholders. Stages 2 and 3 took the form of an action-research process involving a series of face-to-face meetings, email exchanges, observations, and training sessions.

**Results:**

Utilising the three-stage framework, we co-produced and tested intervention content and delivery methods for the two interventions over a period of 18 months involving external partners. New and adapted intervention activities, as well as refinements in content, the format of delivery, timing and sequencing of activities, and training manuals resulted from this process. The involvement of intervention delivery staff, participants and teachers shaped the content and format of the interventions, as well as supporting rapid prototyping in context at the final stage.

**Conclusions:**

This three-stage framework extends current guidance on intervention development by providing step-by-step instructions for co-producing and prototyping an intervention’s content and delivery processes prior to piloting and formal evaluation. This framework enhances existing guidance and could be transferred to co-produce and prototype other public health interventions.

**Trial registration:**

ISRCTN14415936, registered retrospectively on 05 November 2014.

**Electronic supplementary material:**

The online version of this article (10.1186/s12889-017-4695-8) contains supplementary material, which is available to authorized users.

## Background

There are a range of approaches to public health intervention development [1–12]. The UK’s Medical Research Council (MRC) guidance, the most widely cited approach, recommends that intervention development should consist of theory development, identification of an evidence base (typically through a recent or new systematic review), and modelling of processes and outcomes [13]. Other approaches provide more detailed guidance on: developing intervention or program theory [2, 6, 9]; using mapping techniques to inform the components required in an intervention [1, 5, 7, 10]; cycles of testing and refinement [3, 8] and the use of partnerships with individuals, communities, and service providers [4, 7, 8, 12]. These guidelines support development of a theoretical rationale for an intervention, but provide scant pragmatic instruction on how to develop intervention materials and delivery methods.

Theory needs to be translated into intervention design in a way that facilitates adoption across settings and maximises implementation. The RE-AIM framework helped to re-focus away from efficacy to effectiveness, and assess the degree of reach, adoption, implementation and maintenance of effects [14]. As well as identifying reasons for (in)effectiveness, an assumption is that barriers to adoption, implementation and maintenance that are identified in evaluations are addressed in the adaptation of existing or design of new interventions. It is not clear whether this occurs. Even if barriers are addressed, as the policy and practice landscape can change with country, health system and time, some barriers identified may not be relevant in a new system. A method for the rapid identification of potential barriers to effectiveness, possible solutions, testing and re-testing of materials would save the costly implementation of interventions that do not adequately account for variations in context. The involvement of customers in the prototyping of new products has long been used in manufacturing [15], as a method for gaining feedback and improving design. Intervention design may benefit from incorporating the principles of iterative product development and testing intervention components, or prototyping, with those who deliver and receive interventions [16].

The concept of Transdisciplinary Action Research (TDAR) [12] has been developed to support effective collaboration between behavioural researchers, policy makers, frontline public services staff and communities. Building on Lewin’s [17] concept of ‘action research’ that combines scientific and societal value, TDAR is an approach where researchers from multiple disciplines work with a range of stakeholders and intended beneficiaries to jointly understand social problems and identify practical solutions to them, such as through co-producing new public health interventions [5]. A key component of this approach to applied social science is the development of sustainable, replicable processes to support effective collaboration between researcher teams, frontline practitioners and communities in order to harness the latent expertise of key stakeholders (for example, those who deliver health promotion to the target population, gatekeepers within settings such as school teachers, managers, owners) so that the acceptability and feasibility of the intervention is addressed and maximised at the development stage [5, 12, 18–20].

We present the framework for co-production and prototyping which was used to guide the adaptation of the ASSIST smoking prevention intervention to develop detailed content and delivery processes for two new peer-led drug prevention interventions, one as an adjunct to the ASSIST intervention (+Frank) and the other a standalone drug prevention intervention (Frank friends).

### Case study: ASSIST + Frank intervention development study

Informed by the principles of TDAR, we tested a novel, staged approach to adapt and co-produce with stakeholders the content and delivery of two new informal, peer-led interventions to prevent illicit drug use among secondary-school students in the United Kingdom by adapting an effective peer-led smoking prevention intervention (ASSIST) [21]. ASSIST is a school-based peer-led intervention that has been shown to be effective in reducing the uptake of smoking in UK secondary schools [21]. It is recommended in the National Institute for Health and Care Excellence (NICE) guidance on school-based smoking prevention [22] and forms part of the Welsh and Scottish Governments’ tobacco harm reduction plans [23, 24]. In contrast, studies of the implementation and effectiveness of peer-led drug prevention interventions report mixed evidence [25–27]. For example, very low-levels of implementation occurred in the EU-Dap trial, where only 8% of centres implemented all seven peer-led sessions and 71% did not conduct any meetings at all [26]. Moreover, there is some evidence of harmful effects for school students with drug using friends from the US TND-Network trial [27]. These challenges suggested new approaches were warranted and more careful intervention development was required.

Informed directly by the existing evidence surrounding the effectiveness of the ASSIST intervention [21] and its basis in the theory of Diffusion of Innovations [28], we adapted the ASSIST model of informal peer-led delivery (see Additional file 1: Table S1 for components of ASSIST) to drug prevention using information from the UK national drug education website, Talk to Frank [29]. The theoretical basis and design of the effective ASSIST intervention informed a skeleton structure of core intervention components and processes that underpinned the development of the two new informal, peer-led interventions to prevent illicit drug use. From this, an intervention logic model was constructed for each of the two new peer-led drug prevention interventions, +Frank and Frank friends (see Additional file 1: Figs. S1 and S2), which would be compared at subsequent stages of evaluation.

The intervention “+Frank” is as an informal peer-led drug prevention adjunct to the ASSIST smoking prevention intervention. It is designed to be delivered in secondary schools to year 9 students (aged 13–14) who have previously received ASSIST in year 8. “Frank friends” is a stand-alone, informal drug prevention intervention. It aims to identify and recruit peer opinion leaders in year 9 to be trained as peer supporters. Both interventions involve off-site training to learn the effects and risks associated with specific drugs and potential harms; +Frank involves one day and Frank friends two days of training. Peer supporters are asked to have conversations with their peers on the risks of different drugs and log these interactions over 10-weeks. +Frank peer supporters are visited three times and Frank friends four times by trainers to support them to have conversations.

## Methods

A three stage multi-method framework was tested to co-produce the content, resources, and delivery methods for the +Frank and Frank friends interventions based on their logic models. The three stages are: 1) Evidence review and stakeholder consultation; 2) Co-production; and 3) Prototyping. The methods used at each stage allowed for integration of scientific literature with stakeholders’ knowledge and expertise. The key stakeholders in intervention development were the Public Health Wales (PHW) ASSIST delivery team, secondary school students, and health professionals working for drug agencies and with young people. The objectives of the methods used and topics explored at each stage are summarised in Additional file 1: Table S2.

### Stage one: Evidence review and stakeholder consultation

In stage one, ‘evidence review and stakeholder consultation’, members of the research team engaged in a process of co-operative enquiry with stakeholders. A variety of consultation methods were offered to groups of stakeholders to enable them to participate in the way that they felt was most appropriate. The overall aim of the stakeholder consultation was to gather multiple perspectives about drug use issues relevant to young people, existing drug education for young people, and ideas for appropriate and acceptable content for the peer-led drug interventions. This involved a range of methods.

#### Focus groups with young people

Six focus groups were conducted with 47 young people aged 13–15 who were purposively sampled from a range of settings allowing for variation in demographic backgrounds and existing experience of drug use (three schools, a youth centre and a student referral unit). A semi-structured topic guide was used consisting of broad open-ended questions relating to participatory task-based activities using information and resources from Talk to Frank.

#### Interviews with the ASSIST intervention delivery team

Interviews were conducted with an opportunity sample of five members of the PHW ASSIST delivery team.

#### Observations of current practice

Observations of all five stages of ASSIST intervention delivery were conducted (*n* = 8) as well as one observation of the ASSIST ‘Train the Trainers’ course.

#### Stakeholder consultation

A range of unstructured consultations were also conducted with opportunity samples of young people and practitioners: one with five volunteers from a young people’s public involvement group (ALPHA) aged 16–19 years old; one with seven young people aged 13–15; one with five recipients of ASSIST aged 12–13; and nine individual consultations with health professionals working for drug agencies (*n* = 4) or with young people (*n* = 4) or both (*n* = 1).

Audio recordings of the focus groups and interviews were transcribed verbatim and analysed using thematic analysis. An a priori coding framework focused on the objectives of the interviews with the delivery team was applied to the interview transcripts to organise data for subsequent searches for recurring patterns and themes. However, an element of flexibility was maintained such that codes which did not fit the framework were also captured. This analysis approach has been described in detail elsewhere [30].

Researcher field notes from observations and informal consultations were collated and combined with the outcomes from the analysis of interview and focus group data in order to identify similarities and differences across the various stakeholder perspectives emerging from the consultation process. These outcomes were then taken forward to feed into the co-production of intervention content during stage 2.

### Stage two: Co-production

In stage 2, ‘co-production’, an intervention development group consisting of members of the research team and key stakeholders was established to co-produce the intervention materials and resources. The key stakeholders identified for adapting the ASSIST intervention to deliver information from Frank were members of the Public Health Wales ASSIST delivery team. The PHW team had delivered ASSIST to over 350 schools over a period of seven years so had extensive experience of intervention delivery within schools and were well placed to consider the potential feasibility of adapting intervention content for use with an older age group and for drug prevention. The team had also been identified to deliver the new drug prevention interventions that were being developed.

Co-production of intervention content took the form of an action research cycle over a series of meetings of the intervention development group in which findings from stage 1 were considered, ideas were presented by all members, feedback on ideas sought, refinements made and presented again, until final content was agreed. Five face-to-face meetings were held over the course of a four month period. These were supplemented by communications via email where face-to-face meetings were not possible, or when matters arose that required discussion between meetings.

### Stage three: Prototyping

In stage 3, ‘prototyping’, the draft intervention manuals and associated resources underwent expert review by the lead author of the ASSIST randomised controlled trial [21], and the lead trainer of DECIPHer Impact, the company that licenses ASSIST. Reviewers looked over the adaptations made to ASSIST intervention content and resources, as well as newly developed content, and were asked to provide feedback regarding key uncertainties identified during development (for example, fit with the Diffusion of Innovations theory, age-appropriateness of activities, suitability of timings and sequencing).

In order to gain preliminary feedback regarding acceptability and feasibility of the intervention content, intervention delivery was tested with an opportunity sample of the ALPHA group (*n* = 5), as well as during two training sessions with the intervention delivery team. Independent observations of intervention delivery in two test schools were made by two members of the research team using a structured observation tool to check whether the learning outcomes for each activity were met and any deviations that were made.

## Results

**Fig. 1:**
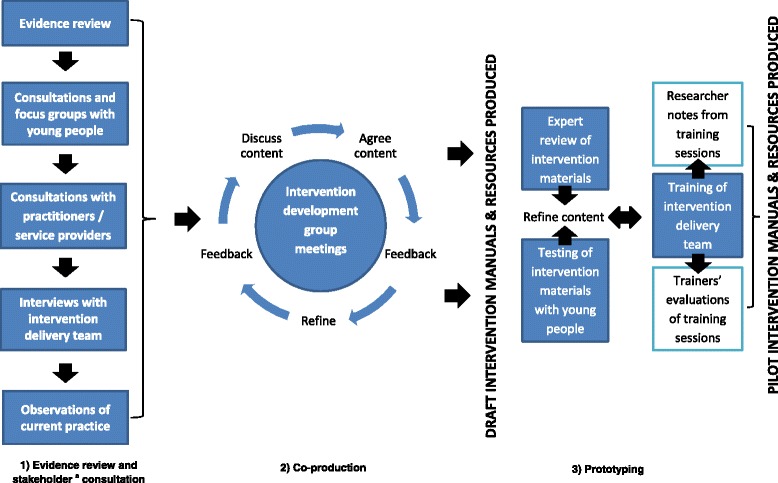
Framework for intervention co-production and prototyping. ^a^ Stakeholders comprise those within or external to the delivery setting (e.g. school-based: school teachers, head teacher, contact teacher, head of Personal, Social, Health and Economic (PSHE) education, head of year, receptionist; national and local policy leads; parents/ guardians/ caregivers)

Figure 1 shows the framework and activities that were completed in the ASSIST + Frank intervention development study stage. The process of co-production and prototyping took 18 months and comprised 42 activities (Fig. S3 shows the frequency and time line of each activity). The process was iterative and cumulative with refinements occurring prior to the next stage.

### Stage one: Evidence review and stakeholder consultation

In line with the MRC guidance on developing complex interventions [13], we reviewed the existing literature to identify the existing distribution of illegal drug use amongst young people and whether there were any existing effective school-based drug prevention interventions. A non-systematic review of population-based prevalence studies with secondary school-aged children in the United Kingdom (aged 11 to 16 years of age) showed the lifetime prevalence of any illegal drug use doubled from 6.8% to 12.4% then 23.1% between the ages of 13, 14, and 15 years respectively [31], this informed our decision to deliver the intervention to UK year 9 students (13 to 14 years of age) as it an age of rapidly increasing drug experimentation. A systematic review of school-based drug prevention found small effects on cannabis use in the short term and poor implementation of interventions that were peer delivered [25, 26]. The development of the +Frank and Frank friends interventions was informed by the effectiveness of the ASSIST intervention [21] and its basis in the theory of Diffusion of Innovations [28], an approach not previously used in relation to youth drug prevention.

During stage one we consulted with key stakeholders with the aim of gathering information to tailor the interventions to the target context and population in order to maximise acceptability and reduce problems with implementation. Key stakeholders were identified as people with direct experience or knowledge of youth drug taking, recipients of the existing ASSIST smoking prevention intervention, intended recipients of the newly developed interventions, and those who delivered any existing drug prevention interventions within the setting (i.e. schools) or provided intervention resources (e.g. financing, staffing).

Table 1 summarises the results from the stage one focus groups, interviews, consultations and observations. Several narratives were replicated across the different stakeholders. With regard to which drugs the intervention should prioritise, the young people aged 12–18, practitioners working in drug support agencies with young people, and the review of prevalence studies all highlighted the same eight drugs which had over a 1% prevalence in 13–15 year olds [31]. The consultations with young people and practitioners also noted a local issue with steroid use in older age groups, which was not apparent in prevalence data as these were gathered in England and did not sample from Welsh schools. These consultations led us to tailor the intervention to the local context by including information on steroids in the interventions.

**Table 1:** Results from application of the 3-stage framework for co-production and prototyping in the ASSIST + Frank study

Activity	Objectives	Results
Stage 1: Evidence review and stakeholder consultation		
Evidence review	Identify target age group for interventions and identify target drugs to focus intervention content on.	• The Smoking, Drinking and Drug Use survey in Young People showed the use of any drug in the last year almost doubled from 6.8% at age 13, to 12.4% at age 14, and then again to 23.7% at age 15; largely due to increases in the use of cannabis [31]; • Target intervention at 13–14 year olds (Year 9 students); • Focus intervention content on drugs with >1% prevalence in 13–15 year olds (cannabis, volatile substances, ecstasy, poppers, cocaine, ketamine, mephedrone, and magic mushrooms).
Consultation with young people’s involvement group	Explore thoughts about drug education in school, their conversations about drugs with friends, awareness of Talk to Frank and opinions of the website.	• Drug education is typically didactic and should be more interactive; • Discussions with peers about drugs are frequent; • Commonly used drugs at their age are alcohol, cannabis, poppers, mephedrone, ketamine and cocaine; • Talk to Frank was viewed positively, but should be accompanied by other visual resources.
Consultation with Year 9 students	Explore views about drug use in their age group and ideas about content for a drug prevention intervention.	• Content suggested included effects of drugs on the body, and the legal consequences of drug possession; • Specific drugs to focus content on included cannabis, alcohol, steroids, magic mushrooms and legal highs.
Focus groups with Year 9 students	Explore knowledge and risk perceptions of drug use and perceptions of drug use prevalence in their age group. Explore acceptability and age-appropriateness of drug education messages on Talk to Frank website.	• Health risks of cannabis are known; • Legal consequences of cannabis use are less well known; • Content on impact of drug use on educational achievement directly, or through school exclusions if caught in possession needed; • Content on impact of drug use on parents worrying about harms (to health, criminal sanctions, schooling exclusions), shame brought to family, and increasing stress would be welcomed; • Attention to potential iatrogenic effect of Talk to Frank messages on amphetamine use promoting weight loss required.
Consultations with stakeholders (Drug agencies and professionals who work with young people)	Explore awareness of drug education resources and support, and views on appropriate content for a drug prevention intervention.	• Cannabis and alcohol are the most commonly used drugs by 13 to 14 year olds; • New Psychoactive Substances (NPS) are an increasing concern, particularly synthetic cannabinoids; but not in 13 to 14 year olds; • Staff from drug agencies noted a local problem with anabolic steroids regarding attendance at needle exchange programs. Use is not in 13–14 year olds; • Existing drug education for 13–14 year olds is either provided in classroom-based sessions, or one-off workshops delivered by a specialist agency or a community police officer; • There are limited drug education resources available and existing resources such as ‘drugs box displays’ are expensive. Resources require regular updates in response to emerging NPS and changing trends.
Consultations with Year 8 recipients of ASSIST	Explore ideas about peer supporter training and content for a drug prevention intervention.	• Content suggested included effects of drugs on the body, how drugs cause ‘highs’, health risks, legal consequences, and harm minimisation; • Specific drugs to focus content on included cannabis, solvents, magic mushrooms, cocaine, speed, mephedrone, legal highs, and steroids.
Observations of current ASSIST practice	Identify aspects of the intervention that work well and could be adapted for use to deliver a drug prevention intervention and with a Year 9 population.	• Flexibility in adapting timings and delivery modes to respond to student engagement is key for successful delivery of training; • Need for clear objectives noting which are essential to deliver.
Interviews with intervention delivery team	Identify possible influences on intervention feasibility and acceptability. For example, explore aspects of ASSIST that could be adapted for use to deliver a drug education intervention and for use with 13–14 year olds, as well as those which might not lend themselves to adaptation.	• Intervention activities need to be interactive; • Successful implementation of intervention requires flexibility in delivery to meet needs of different groups; • Some intervention activities required updating (e.g. ASSIST activity using postcards because peers supporters did not know what they were); • Some intervention activities might be too immature for use with 13–14 year olds; • Delivery of messages about harms of drug use is much more complex than harms of smoking (more compounds with different effects); • Concerns around amount of knowledge required to deliver drug prevention intervention.
Stage 2: Co-production		
Meetings of the intervention development group	Action research cycle of assessment, analysis, feedback and agreement on the core components of the intervention required to educate peer supporters on the harms of drug use and the skills required to communicate these to their peers.	• Findings from Stage 1 suggested long-term harms to health of low-levels of cannabis are less definitive than those of smoking; • Include content on concerns expressed by young people and harms associated with drug use that they did not know about; • Shift focus towards these concerns and away from harms to health of the most commonly used drug - cannabis; • Highlight the potential immediate harms to health from use of glues, gasses and aerosol (i.e. sudden sniffing death); • Harms associated with drugs being unregulated and illegal: unknown compound and dose, thus unexpected effects are likely; • Potential consequences of sanctions imposed by schools (temporary, permanent exclusion) and poorer educational achievement; • Potential consequences of criminal sanctions on travel and future career options; • Mention harms including increasing parental anxiety, stress and shame; • Draft intervention manuals and associated resources detailing intervention activities and how these should be delivered were produced.
Stage 3: Prototyping		
Expert review of intervention materials	Identify potential problems or weaknesses in intervention materials prior to piloting.	• Updating of some intervention activities was welcomed; • More detail needed in instructions for delivery team; • Refining of timings for some intervention activities.
Testing of intervention materials with young people	Delivery of intervention. Identification of issues around feasibility and acceptability of newly developed intervention content.	• Intervention activities were well received; • Refinements included amending wording, providing more detailed instruction and objectives, and using smaller groups.
Training of intervention delivery team	Simulation of intervention delivery. Identify issues around feasibility and acceptability of intervention content.	• Need for additional drug education training; • Refinements included amending timings, clarifying ambiguities in instructions, changing format of delivery, adding extra content and removing content.

The consultations and focus groups with young people suggested that 13 to 14 year olds were relatively familiar with the potentially harmful effects of drugs on health.

“*Like we all know weed is bad, we all know what it does to you as well*.” *[P1, male]*

Young people were less familiar with the potential legal consequences of being caught in possession of an illegal drug in the UK.

*“When it says unlimited fine, does that mean the police can just charge you?” [P2, female]*

The familiarity of young people with the harms of drugs on health, prompted us to also add focus on the harms associated with drugs being illegal and therefore unregulated, such as unexpected effects brought about by consuming an unknown compound of an unknown dose. Other concerns that young people voiced included the potential harms of drug use on family relationships, future education and employment.

*“I mean that’s your mum, that’s one of your parents, they put a roof over your head. If you get drove away from them you don’t get food for yourself, you don’t get a roof over your head, you’re out on the streets. You don’t have anyone to get you a meal or look after you ‘cause you’re on your own.” [P3, male]*

*“‘Cause then you’re getting a criminal record that’s stopping you from getting a job and loads of stuff.” [P4, female]*

A number of factors that might influence the engagement of students during peer supporter training were found in both the interviews with the ASSIST delivery team and independent observations by the research team of delivery of the intervention. In particular, the importance of flexibility in delivery of intervention activities to different groups and the need for engaging, interactive content.

*“We work to the same objectives, but in terms of how we run some activities, we might change them a bit … with different groups you know, how they react to a certain activity you might change it round to help the running of it.” [T1]*

*“I think it’s important that whatever we do that it’s quite engaging and [students] get an input as well, you know, not just sitting there watching us, listening to us, I think it’s important that it’s interactive as well.” [T3]*

*“Making sure that they’re interactive … so they’re up and about, they get moving around, break off activities, um, just making it as interactive as possible.” [T4]*

### Stage two: Co-production

During the co-production process, the intervention development group reflected on findings from stage one and used these to inform the adaptation of content from ASSIST and the development of new content. The group was participatory and collaborative and all members were provided with opportunities to input. This process exploited the intervention delivery team’s experience with the setting, target population, and intervention content. For example, it was noted during the stage 1 interviews with the ASSIST team that an important aspect of the intervention for them was providing the peer supporters with interesting and memorable facts about smoking that they could use in conversations with their peers.

*“There’s key facts within ASSIST … four thousand chemicals [in a cigarette], um, sixty to seventy chemicals cause cancer, and we always get the impotence one as well. So the boys always remember the erectile dysfunction.” [T3]*

*“So if we can give them facts that sort of link into what they could be talking about with their friends, it makes it easier for these conversations to happen. In ASSIST, one of the facts they always remember, is that smoking could affect your ability to get an erection. That is the one that sticks with them, and you might not have done the training for ten weeks, and they will still remember that.” [T1]*

*“In ASSIST, we know that young people will leave knowing the ingredients of a cigarette, long-term, short-term health effects, is it guaranteed. We know that you'd go up to any young person that had done the training and you'd ask them how many ingredients are in a cigarette and they’d be able to tell you.” [T1]*

This was also observed in field notes of the observations of delivery of the ASSIST intervention made by the research team. During stage two, the intervention development group considered these findings and decided to adapt information from the Talk to Frank website [29] about the risks of drug use into memorable factual statements. These key statements were then used across several activities within the peer supporter training and added to the peer supporter diaries as a reminder. Examples of the statements include; “Cannabis contains some of the same chemicals as tobacco”, “A drugs-related conviction can stop you from travelling to some countries, such as the USA” and “Giving cannabis to your mates is considered ‘supplying’ under the law”.

Both the research and ASSIST teams independently developed adaptations and new content which were shared amongst the group. For example, a member of the ASSIST team had already developed a new mode of delivery for one of the training day activities in ASSIST in order to address an existing feasibility issue. This was incorporated into the adapted activity for the new interventions.

### Stage three: Prototyping

A period of prototyping of the intervention content, materials and delivery methods is a necessary next step for identifying early issues with acceptability, feasibility and other potential teething problems so that these can be addressed prior to formal piloting and evaluation.

Expert peer review of intervention content or components is useful for examining key uncertainties that have been identified during development. Expert reviewers should be selected based on the areas of greatest of uncertainty and be independent of the intervention development group. There were two areas of uncertainty identified during the development of +Frank and Frank friends; how newly developed activities fit with the diffusion of innovations theory, and whether the format of activities was age-appropriate and followed suitable timings and sequencing.

We sought expert feedback from the lead author of the ASSIST RCT [21] to examine fit with theory and from the lead trainer at DECIPHer Impact who delivers all training to new ASSIST teams to advise on timings and sequencing. The feedback received included possible minor refinements to the timing of some intervention activities and the presentation of instructions in the intervention manual. In addition it was suggested that consideration was given to ‘future proofing’ intervention resources by identifying content that may require regular review and updating.

Testing delivery of the draft intervention content or components on a small scale is also recommended. Where possible the intervention should be delivered to a sample of the target population, if not it is advisable to make use of opportunities for simulated delivery. During testing, data should be collected to explore the experiences of those delivering the intervention as well as those receiving it in order to inform refinements.

Table 2 shows an example of how intervention content was co-produced over each stage of the framework, including how the iterative process of gathering feedback and making refinements was made during the prototyping stage in response to delivery of an activity from the peer supporter training. The objective of the activity (titled ‘What is a drug?’) is to define, name and categorise the effects of drugs. A series of insights were generated from testing out delivery with a group of young people, as well as during training of the intervention delivery team, where the trainees practised delivery of intervention activities with each other. Without this period of testing, these issues would not have emerged until formal piloting. These included: trainers being anxious they would have to have an encyclopaedic knowledge about drugs after young people generated over thirty names of drugs in the test phase; underestimating the time taken to list drugs during the activity; and confusions over drugs with a dual effect. Refinements were made to the training manuals and activities were amended to address these findings and the content was tested again.

**Table 2:**
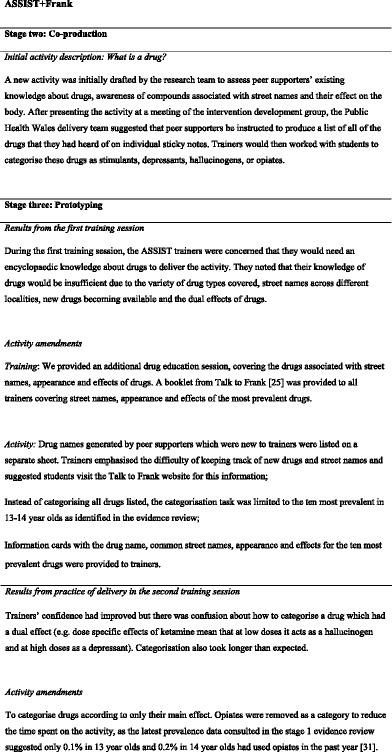
Example of co-production and prototyping of intervention content in ASSIST+Frank

### Reflections on co-production

Interviews with the ASSIST team at the end of stage three suggested they believed co-production created a sense of ownership and buy-in of the intervention, which they were going to be delivering as part of the study:

*‘Oh I really enjoyed it, I think it was very beneficial, especially because if, we’re the ones that’ll be ending up delivering it’ [T1]*

*‘It’s good that you know, I can say that I’ve kind of contributed towards developing something new’ [T2]*

*‘. . . The team appreciate being asked as well because you know in the future if we are expected to deliver, knowing that we’ve been part of it from the start really does help*’ *[T4]*

Throughout co-production the intervention delivery team had been able to convey the realities of delivering interventions to young people and had highlighted important potential barriers to implementation which were addressed at an early stage.

‘*I think it’s helped to have us involved, just because we’ve got the hands-on experience of working with young people’ [T5]*

Independent observations by the research team of delivery of the finalised intervention identified that some trainers continued to adapt activities during delivery, after co-production had ended. In the +Frank intervention, across the 15 activities, five were delivered in full, eight had minor deviations from the manual and two were not delivered at all. In the Frank friends intervention, across the 25 activities, 13 were delivered in full, nine had minor deviations and three were not delivered at all. Field notes suggested the delivery team struggled to switch off from an intervention development mind-set even after co-production had ended. If carried through to formal piloting, the interventions may not be delivered entirely as intended which may potentially be a barrier to implementation with high fidelity.

## Discussion

The three-stage framework presented extends current guidance by providing pragmatic guidance on how to co-produce and prototype public health intervention content and delivery methods before formal piloting. It provides a framework to guide co-production with stakeholders so that intervention content is tailored to the population and setting in order to address implementation issues at the design stage. This is complementary to existing intervention development guidelines which provide information about the use of mapping techniques [1, 5, 7, 10], intervention theory development [2, 6, 9] and testing [3, 8]. Our framework offers insight into how collaboration and co-production with stakeholders can be incorporated into these stages of intervention development.

The incorporation of stages of co-production and prototyping builds on existing literature on Transdisciplinary Action Research [5, 12] as well as theories of capacity building noted in community psychology [32], participatory action-research [33], plan-act-study-do cycles in clinical settings [34, 35], and the use of quality improvement replications to improve systems [36]. The involvement of key stakeholders in the co-production of intervention content provides a mechanism for tailoring intervention content to the context and target population to maximise acceptability and reduce the likelihood of problems with implementation. A variety of stakeholders should be engaged to ensure that a range of expertise and perspectives relevant to the realities of the intervention problem, target population, and intended delivery setting is represented.

The case study presented here provides an example of co-production with key stakeholders throughout the lifecycle of intervention development to adapt content from an existing effective peer-led smoking prevention intervention to co-produce two new peer-led drug prevention interventions. Based on this experience we offer some reflections on the benefits and potential weaknesses of such an approach.

### Benefits of co-production

The involvement of stakeholders with knowledge and experience of existing interventions, the target population, and the delivery setting has the purpose of maximising the acceptability, feasibility and quality of the intervention being developed and its fit with the implementation context. For example, frontline practitioners know the delivery setting, as well as issues that have affected the implementation of previous interventions. In addition, co-production engenders an element of ‘buy-in’ to the intervention and creates a sense of ownership amongst those involved in its development. This can be particularly useful where the intended intervention deliverers can be identified at the development stage and invited to be involved in the intervention development process. In addition, the involvement of the intended intervention recipients during co-production can help to ensure that intervention content meets their needs and is acceptable and credible.

### Weaknesses of co-production

The co-production process is both iterative and fluid. However, there must come a stage in the process where intervention content is consolidated and put to the test. Observations of delivery found that some staff made amendments to activities, after it was agreed that co-production had ended and the intervention manual finalised. This meant that out of 40 activities 17 (42.5%) were delivered with a minor deviation from the instructions in the manual and five (12.5%) were not delivered at all. This suggests it may be difficult for stakeholders to demarcate when the co-production process has ended, which may be a threat to fidelity if carried through to formal delivery outside of piloting.

There are several potential barriers to co-production including competing priorities and goals and interdisciplinary conflict between the stakeholders involved in the intervention development process. This is more likely when the stakeholders involved are from a range of background fields, bridging both professional and lay perspectives [12]. Another potential barrier is the time consuming nature of co-production which requires active engagement from those involved over an indeterminate amount of time to allow the process to unfold and evolve. Some stakeholders may not have the flexibility within their roles that the PHW ASSIST team had so may not be so heavily involved. There may be some potential limits to the transferability of this approach for the development of other public health interventions. The framework was used to adapt an existing intervention with a strong evidence base and a well-established delivery structure. In addition, the PHW ASSIST team were highly experienced in terms of knowledge and delivery of ASSIST to the target population. These conditions may have contributed to the successful application of the framework within this study.

## Conclusions

The framework presented here provides pragmatic instruction on how to coproduce and prototype public health interventions. It complements other intervention development guidance by providing more detail on the process of the early stages of intervention development and co-production that receives limited attention in existing guidance on intervention development [1–12]. Future studies should explore its utility in guiding the process of co-production of interventions with different target behaviours, populations and stakeholder groups.

## Additional file

Additional file 1: Table S1. Core components of the ASSIST intervention. **Table S2.** A checklist for the key components of the framework for coproduction and prototyping. **Figure S1.** ASSIST +Frank logic model. **Figure S2.** Frank friends logic model. **Figure S3.** Gantt chart of intervention development and adaptation. (DOCX 791 kb)

## Abbreviations

ASSIST: A Stop Smoking In Schools Trial
MRC: Medical Research Council
PHW: Public Health Wales
TDAR: Transdisciplinary Action Research
