# Supplementary material for: Development of a framework for the co-production and prototyping of public health interventions
Source: BMC Public Health. 2017 Sep 4;17:689. doi: 10.1186/s12889-017-4695-8 (PMC5583990; doi:10.1186/s12889-017-4695-8)
Supplement: Additional file 1: Table S1. — Core components of the ASSIST intervention. Table S2. A checklist for the key components of the framework for coproduction and prototyping. Figure S1. ASSIST +Frank logic model. Figure S2. Frank friends logic model. Figure S3. Gantt chart of intervention development and adaptation. (DOCX 791 kb) [file 12889_2017_4695_MOESM1_ESM.docx]

**Table S1. Core components of the ASSIST intervention**

| **Nomination of peer supporters** |
| --- |
| Year 8 students are asked to identify influential peers using three questions - “Who do you respect in year 8 at your school?”, “Who are good leaders in sports or other groups activities in year 8 at your school?”, and “Who do you look up to in year 8 at your school?” The 17.5% of year 8 students receiving the most peer nominations are invited to a recruitment meeting. |
| **Recruitment of peer supporters** |
| A meeting is held with nominees to explain the role of a peer supporter and answer questions. The ASSIST trainers make it clear that students who smoke can only be peer supporters if they commit to trying to stop smoking. |
| **Training of peer supporters** |
| The aims of the training are to: provide information about risks of smoking and benefits of remaining smoke-free; develop communication skills including, listening, cooperation and negotiation, and conflict resolution; enhance students’ confidence, empathy, assertiveness, attitudes to risk-taking, and exploration of personal values and to role play having informal conversations about smoking with their peers. Training takes place at a venue outside school over 2-days and is delivered by a team of external trainers experienced in youth work and health-promotion. |
| **Intervention period** |
| 8-10 week peer-led intervention where peer supporters have informal conversations with their peers about the harms and risk of smoking, when travelling to and from school, in breaks, at lunchtime, and after school in their free time, and log a record of these conversations in a pro-forma diary. Trainers deliver four follow-up school visits to meet with peer supporters and provide them with additional support and training and to review progress with the informal conversations. |
| **Acknowledgment of peer supporters’ contribution** |
| All peer supporters are presented with a certificate to recognise their involvement in the intervention. |

**Table S2. A checklist for the key components of the framework for coproduction and prototyping**

| **Stage 1** | **Evidence review** |
| --- | --- |
|  | - Review literature on effectiveness of existing interventions - Review prevalence data related to the target health issue to identify potential target population |
|  | **Stakeholder Consultation** |
|  | - Identify key stakeholders within the target context and population, including potential intervention deliverers and commissioners - Utilise a variety of formal/informal consultation methods - Explore ideas for intervention content and initial perceived feasibility and acceptability of ideas - Observe current practice if applicable / if adapting an existing intervention |
| **Stage 2** | **Co-production** |
|  | - Establish an intervention development group to consist of members of the research team and key stakeholders identified in Stage 1 - Action research cycle of meetings in which you:  1. Reflect on findings from Stage 1 to inform intervention 2. development 3. Bring ideas for content to discuss within the group 4. Agree ideas to take forward and produce drafts 5. Review drafts and feedback 6. Revise drafts where necessary |
| **Stage 3** | **Prototyping** |
|  | - Identify any key uncertainties that arise during development - Subject intervention content/materials to expert peer review - Select expert reviewers based on areas of greatest uncertainty - Test delivery of intervention content on a small scale - Collect data to explore experiences of deliverers and recipients to inform refinements - Change intervention and collect data - Repeat testing if necessary |

**Figure S1. ASSIST +Frank logic model**
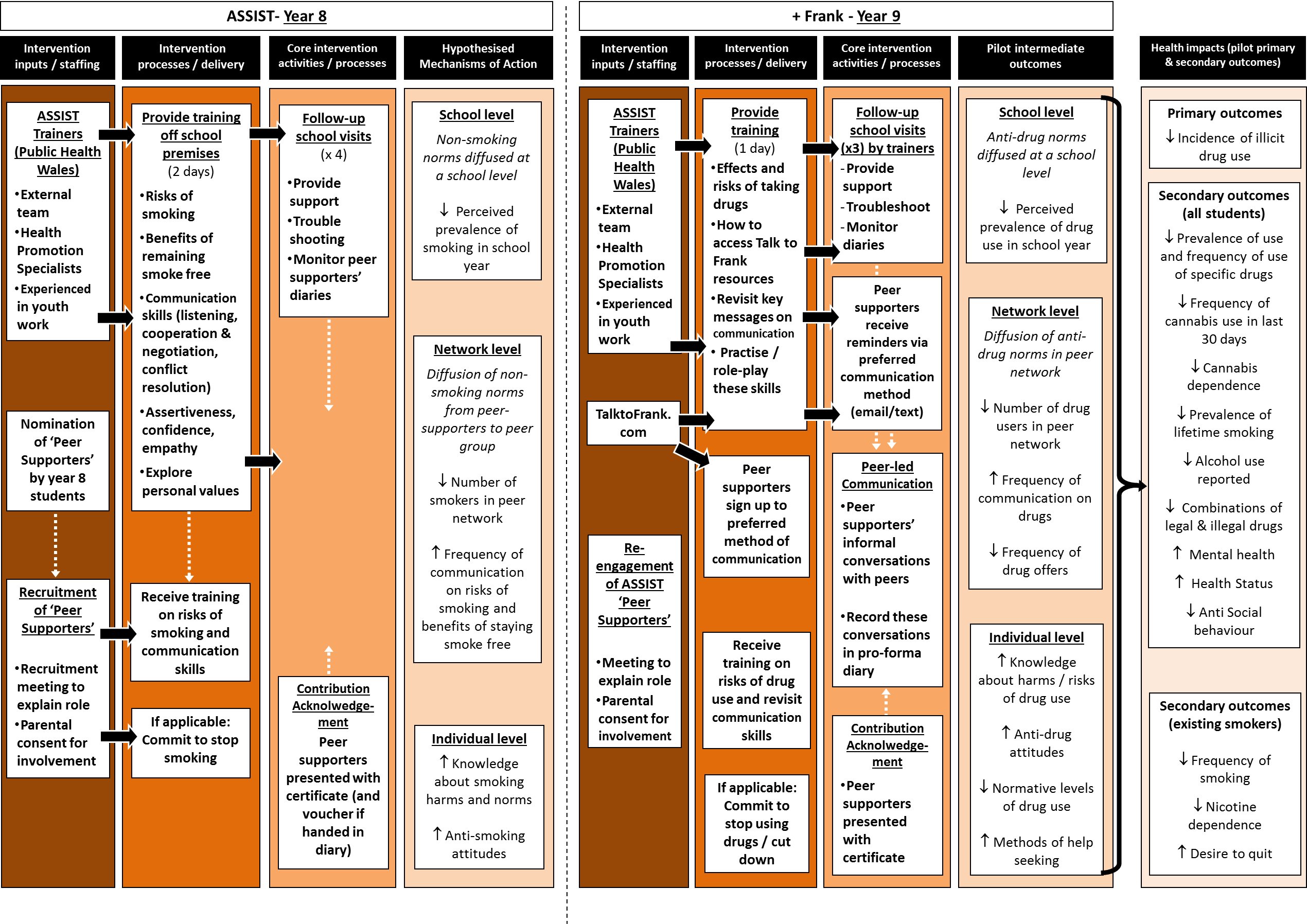


**Figure S2. Frank friends logic model**


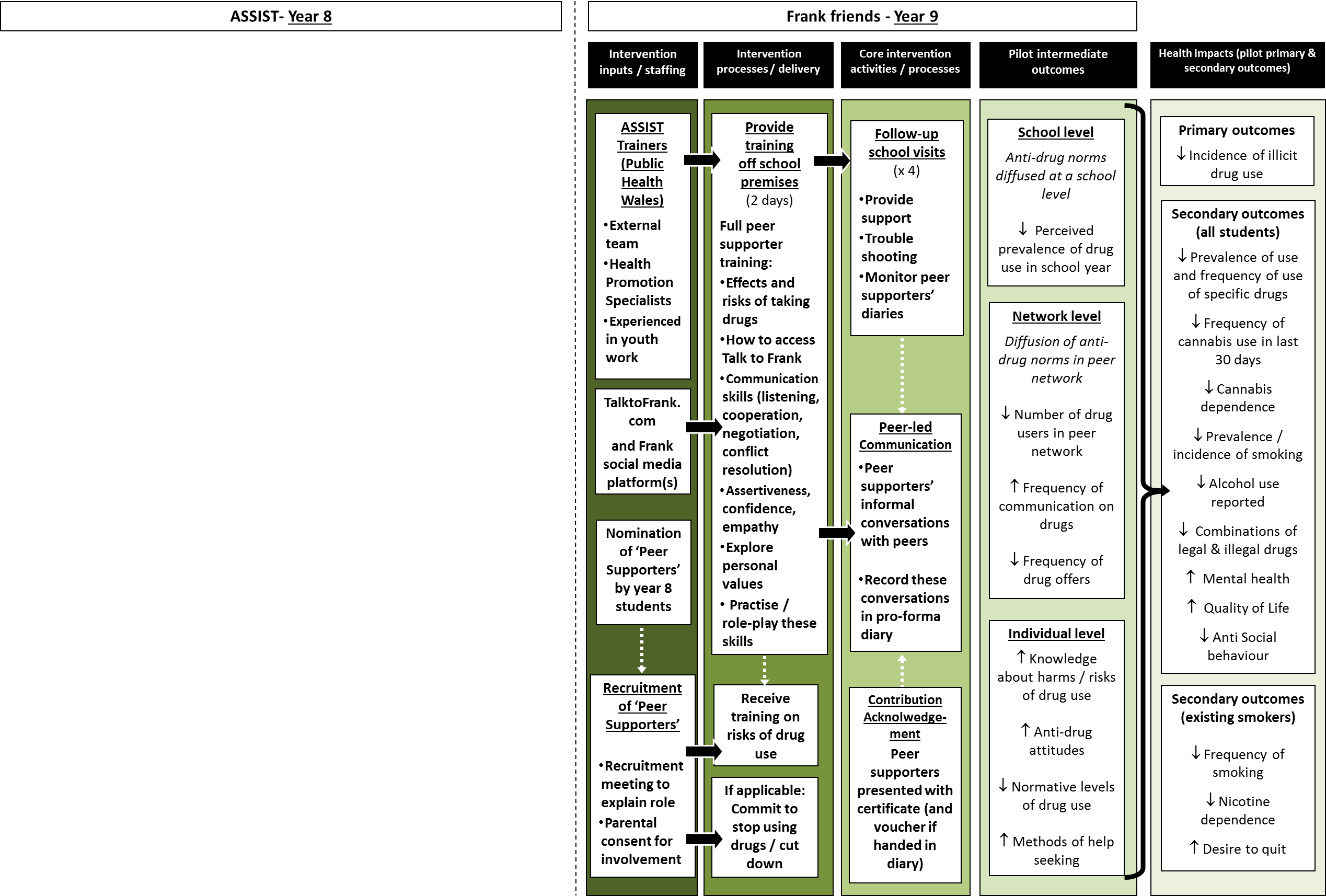


**Figure S3. Gantt chart of intervention development and adaptation**

**
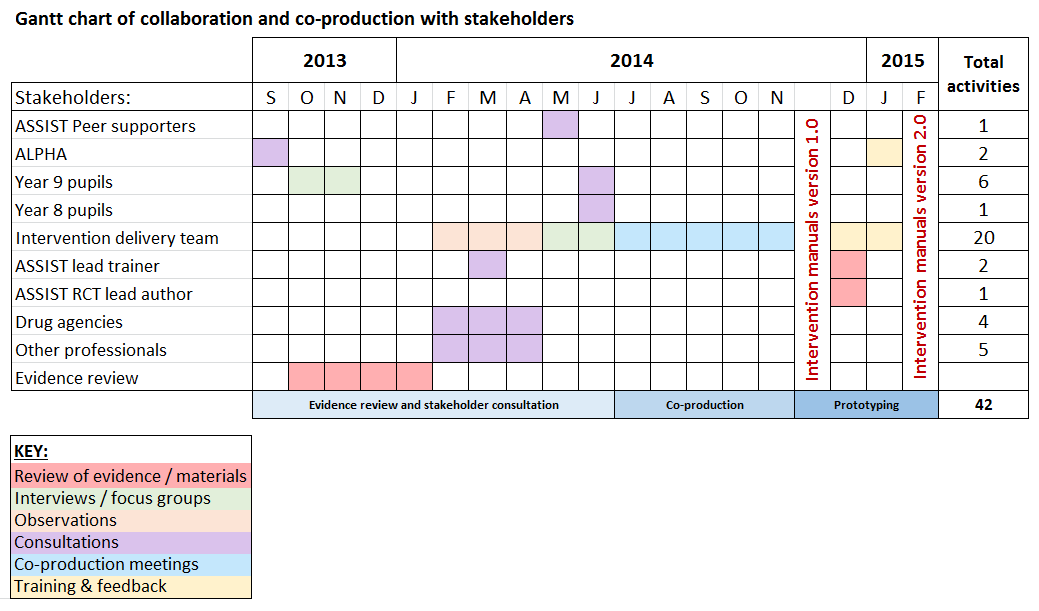
**
